# Outcomes of Endoscopic-Ultrasound-Guided Cholangiopancreatography: A Literature Review

**DOI:** 10.1155/2013/869214

**Published:** 2013-03-18

**Authors:** Shahzad Iqbal, David M. Friedel, James H. Grendell, Stavros N. Stavropoulos

**Affiliations:** Department of Medicine, Division of Gastroenterology, Winthrop University Hospital, Mineola, NY 11507, USA

## Abstract

Endoscopic retrograde cholangiopancreatography (ERCP) can fail in 3–10% of the cases even in experienced hands. Although percutaneous transhepatic cholangiography (PTC) and surgery are the traditional alternatives, there are morbidity and mortality associated with both. In this paper, we have discussed the efficacy and safety of endoscopic-ultrasound-guided cholangiopancreatography (EUS-CP) in decompression of biliary and pancreatic ducts. The overall technical and clinical success rates are around 90% for biliary and 70% for pancreatic duct drainage. The overall EUS-CP complication rate is around 15%. EUS-CP is, however, a technically challenging procedure and should be performed by an experienced endoscopist skilled in both EUS and ERCP. Same session EUS-CP as failed initial ERCP is practical and may result in avoidance of additional procedures. With increasing availability of endoscopists trained in both ERCP and EUS, the role of EUS-CP is likely to grow in clinical practice.

## 1. Introduction

Endoscopic retrograde cholangiopancreatography (ERCP) is the standard procedure for decompression of biliary and pancreatic ducts. Although the success rate is very high, it can fail in 3–10% of cases even by an experienced endoscopist [1, 2]. Percutaneous transhepatic cholangiography (PTC) [3, 4] and surgery [5, 6] have been the traditional alternatives. However, there is morbidity and mortality associated with both. PTC has a complication rate of up to 30% [4]. Although surgery offers long-term patency, it is also associated with increased morbidity as well as mortality [6]. Since first reported by Wiersema et al. [7], endoscopic ultrasound guided cholangiopancreatography (EUS-CP) is now increasingly being employed at expert centers as an alternative to surgery or PTC. 

An online pubmed search was conducted to review the published case reports and series on EUS-CP. The key words used were endoscopic-ultrasound-guided cholangio-pancreatography, endoscopic-ultrasound-guided cholangiography, endoscopic-ultrasound-guided pancreatography, failed endoscopic retrograde cholangiopancreatography, endoscopic-ultrasound-guided therapeutic interventions, endoscopic-ultrasound-guided biliary drainage, and endoscopic-ultrasound-guided pancreatic drainage. All studies and case series involving at least 5 patients were included for the present review. The purpose of this paper was to analyze the published data on EUS-CP and assess its overall efficacy and safety in decompression of biliary and pancreatic ducts. First the indications and techniques of EUS-CP will be discussed, followed by efficacy, safety, and role in clinical practice.

## 2. Indications of EUS-CP

The first case series of EUS-guided cholangiogram was reported by Wiersema et al. in 1996 [7]. Biliary drainage has been performed for both malignant as well as benign indications. The reported malignant biliary indications were pancreatic cancer, metastatic cancer, cholangiocarcinoma, gallbladder cancer, ampullary cancer, and duodenal cancer. Following were the benign biliary indications: bile leak, benign strictures (PSC or iatrogenic), choledocholithiasis, and papillary stenosis. The reported pancreatic indications were pancreas divisum, benign pancreatic duct strictures (chronic pancreatitis, postsevere acute pancreatitis), postsurgical (Whipple) pancreaticojejunostomy stricture, pancreatic stone with obstruction, pancreatic leak ± fistula, and papillary stenosis. The pancreatic duct was dilated (>4 mm in diameter) in most of the studies. However, the nondilated duct was also accessed in recent studies [8]. Initially, EUS-CP was performed on a subsequent day after failed initial ERCP. However, there was a trend towards same day/session EUS-CP with recent studies [9].

In general, EUS-CP can be considered in patients with native papilla after failed initial ERCP or inaccessible papilla due to either obstructed gastrointestinal tract lumen or surgically altered anatomy. The procedure is especially helpful in altered anatomy cases after failed initial ERCP like post-Whipple, Billroth II gastrojejunostomy, hepaticojejunostomy, gastric bypass, and duodenal switch. The bile duct can be accessed by either an extrahepatic or intrahepatic approach. The decision between the extrahepatic and intrahepatic approach is based on the following factors: presence of intrahepatic dilation, presence of gastric outlet obstruction, and ability to reach the second part of duodenum.

## 3. Technique

### 3.1. Patient Selection and Preparation

All such cases should be performed in a tertiary care center by an experienced endoscopist who is proficient in both ERCP and EUS. Repeat ERCP should be attempted on patients referred to the tertiary care center before resorting to EUS-CP. The failed ERCP was defined as failed deep access to bile or pancreatic duct despite the use of advanced cannulation techniques including precut sphincterotomy [10]. The procedure should be done in a dedicated interventional endoscopy room equipped with both fluoroscopy and EUS capability. An informed consent explaining the risk and benefits of EUS-CP versus PTC and surgery needs to be explained to the patient. Prophylactic antibiotics should be administered. Since EUS-CP is a longer procedure, anesthesia assistance should be sought. In the published data, all such cases were done either under intravenous sedation or general anesthesia. It is also important to have back up of both surgical and interventional radiology services.

### 3.2. Instruments and Accessories Selection

The procedure is done using a curvilinear array echoendoscope, preferably therapeutic with working channel of over 3 mm. The following therapeutic echoendoscopes are commonly used in the United States: GF-UCT140 (Olympus America Inc, Center valley, PA, USA) and EG-3870UTK (Pentax of America Inc, Montvale, NJ, USA) with working channels of 3.7 and 3.8 mm, respectively. These allow placement of stents up to 10 Fr (French) in diameter (Table 1).

A 19- or 22-gauge FNA (fine needle aspiration) needle is used for initial duct puncture. A 5 Fr needle knife or 19-gauge fistulotome can also be used for duct puncture. One of the following long (450 or 480 cms) guidewires are then passed into the duct: 0.018 inch, 0.021 inch, 0.025 inch, or 0.035 inch. The 19-gauge FNA needle allows passage of all guidewires, while 22-gauge one allows only 0.018 and 0.021 inch guidewires. It is technically easier to deploy a subsequent stent over a wider diameter guidewire. However, the maneuverability is relatively better with smaller diameter guidewire. The following accessories are used for dilation of newly created fistula in selected cases (especially in transluminal and antegrade stenting): 6–10 Fr bougie (SBDC; (Cook Medical Inc, Bloomington, IN, USA)), 4–6 mm dilation balloon (Boston Scientific, Natick, MA, USA), ERCP 3.9-4.9 Fr sphincterotome (Boston Scientific, Natick, MA, USA), 5.5 Fr Needle Knife cautery (Boston Scientific, Natick, MA, USA), or 6–8.5 Fr Cystotome (EndoFlex, Voerde, Germany). The use of needle knife cautery should be avoided if possible as it was shown to be associated with postprocedure complications in a multivariate analysis by Park do et al. [11]. The rest of the accessories (including stone retrieval balloon and stents) are the same as those for conventional ERCP.

### 3.3. Technical Methods

#### 3.3.1. Biliary EUS-CP

As mentioned before, the bile duct can be accessed by either extrahepatic (transenteric-transcholedochal) or intrahepatic (transgastric-transhepatic) approach. According Maranki et al. [12], the extrahepatic approach is less challenging and should be preferred when second part of duodenum is accessible.

#### 3.3.2. Extrahepatic Biliary Tree

The echoendoscope is positioned either in the duodenal bulb or distal antrum for extrahepatic approach. Color-Doppler US is used to confirm lack of vascular structures. One of the EUS-FNA needles (as mentioned previously) is used to puncture the extrahepatic bile duct. Upon removal of stylet, the fluid is aspirated to confirm entrance of needle tip inside the duct. Contrast is injected under fluoroscopic guidance to obtain a ductogram. A long (450 or 480 cms) guidewire is passed into the bile duct. EUS-CP is then completed by one of the following techniques: ductography, rendezvous with transpapillary stenting, antegrade tract dilation/stenting, and transluminal tract dilation/stenting.

**Figure 1:**
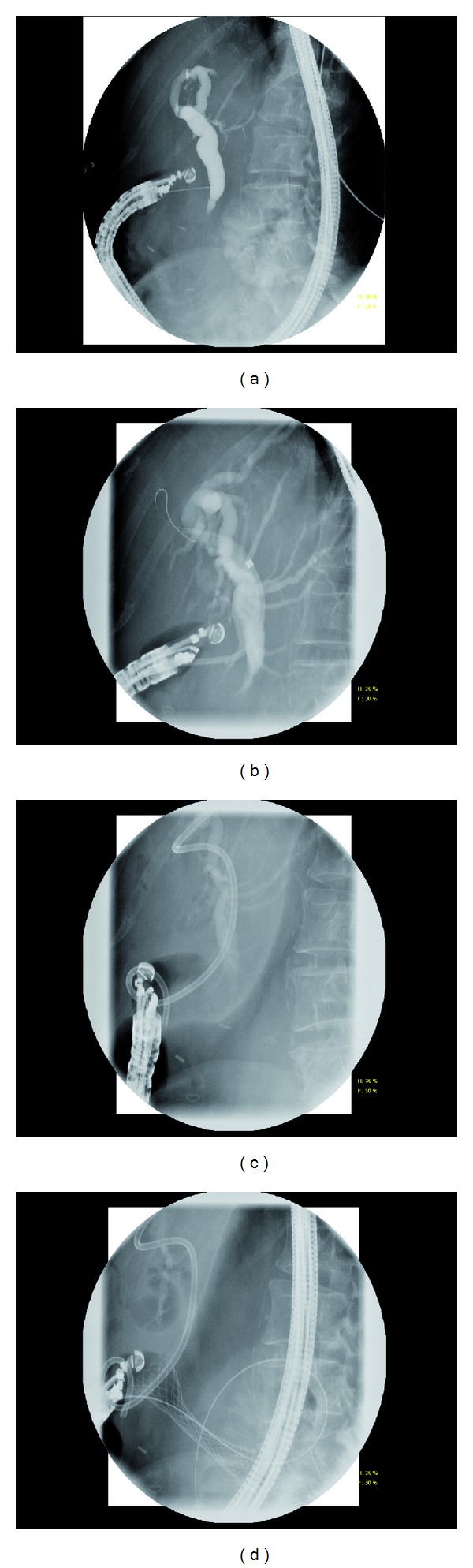
Transluminal stenting in a patient with metastatic breast cancer with extrahepatic biliary and duodenal obstruction. (a) Initial Cholangiogram using 22-gauge needle via transduodenal approach. (b) Choledochoduodenostomy tract dilation with 7–10 Fr dilating catheter. (c) Placement of a 10 Fr × 6 cm double-pigtail plastic stent. (d) Placement of a 22 × 60 mm uncovered enteral stent.

(1) Ductography: after EUS-FNA needle has been passed into the bile duct, contrast is injected. The opacified duct is then used as a guide for retrograde cannulation by a duodenoscope. It may facilitate cannulation by causing visible ampullary bulge in cases with flat intradiverticular papilla [13].

(2) Rendezvous: the EUS-FNA needle tip is oriented in a caudal direction, and attempts are made in passing the guidewire across the papilla. If successful, the echoendoscope is removed leaving the guidewire in place, with the upper end securely held near patient's mouth. A duodenoscope is passed beside the guidewire into the second part of duodenum. The guidewire is caught with a rat tooth forceps or snare and pulled through the operating channel of the duodenoscope. The rest of the procedure is completed in a retrograde ERCP fashion. Instead of catching the guidewire, biliary cannulation can also be done alongside the guidewire by passing another guidewire or sphincterotome next to it.

(3) Antegrade: if transpapillary guidewire passage is unsuccessful or papilla is not accessible, antegrade approach can be attempted. The fistula tract is first dilated (with one or a combination of previously mentioned dilation accessories), followed by antegrade placement of stent across the stricture (and possibly transpapillary, if possible). Antegrade clearance of stones can also be achieved in selected cases. 

(4) Transluminal: the EUS-FNA needle tip is oriented in upward direction, and the guidewire is passed in an upward direction of the puncture. The fistula tract is dilated (with one or a combination of the previously mentioned dilation accessories), followed by transenteric-transcholedochal placement of stent(s). Unlike pancreatic pseudocyst drainage, it is important to focus on EUS and fluoroscopic views rather than endoscopic view during tract dilation and stenting. Only for the final part of stent placement, the echoendoscope is withdrawn to get endoscopic view. For metal stents, sufficient (about 2 cms) intraluminal length is needed to compensate for foreshortening postdeployment. It is our expert opinion that transluminal stenting is more technically challenging than other EUS-CP techniques. However, in cases where the guidewire does not cross papilla and antegrade stenting is not possible due to acute angulation, transluminal stenting is the only possibility (Figure 1).

#### 3.3.3. Intrahepatic Biliary Tree

**Figure 2:**
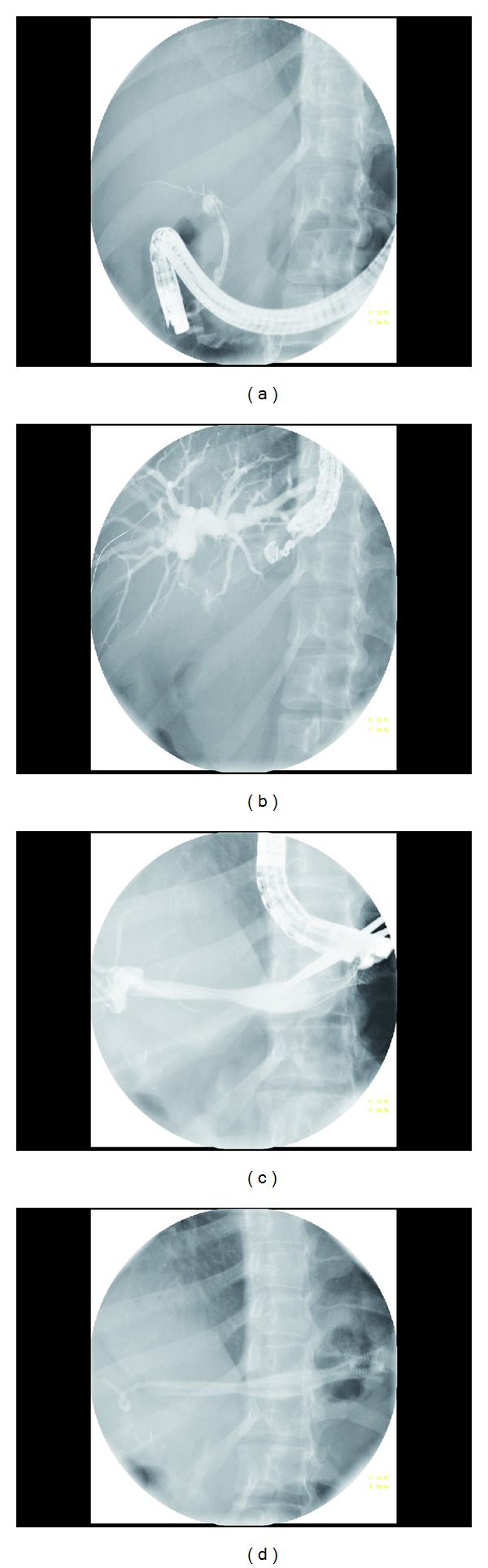
Transluminal stenting in a patient with common hepatic duct transection post-cholecystectomy. (a) Complete iatrogenic CHD obstruction at the site of cholecystectomy clips. (b) Initial cholangiogram with a 19-gauge needle via transgastric approach with passage of 0.025′′ guidewire. (c) Placement of two 10 × 80 mm partially covered SEMS. (d) Placement of a 7 Fr × 12 cm double-pigtail plastic stent inside metal stents to prevent outmigration.

The echoendoscope is positioned in the cardia or lesser curvature of stomach for intrahepatic (left liver) approach. The intrahepatic tree can also be accessed through distal esophagus [14]. One of the EUS-FNA needles (as mentioned previously) is used to puncture the left intrahepatic biliary tree. The rest of the procedure is similar to that described for extrahepatic approach. During transluminal technique, attempts should be made to advance the guidewire either into the right intrahepatic ducts (if possible) or to make few intrahepatic loops in order to provide stability for subsequent tract dilation and stenting (Figure 2).

#### 3.3.4. Pancreatic EUS-CP

**Figure 3:**
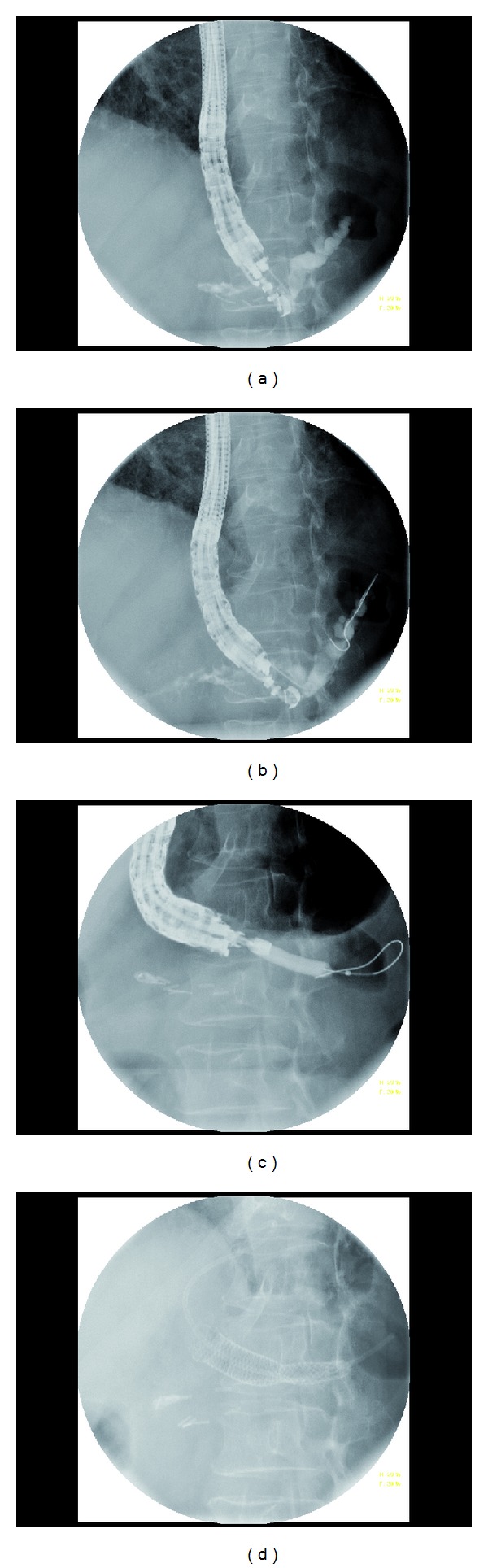
Transluminal stenting in a patient s/p central pancreatectomy with pancreaticogastrostomy obstruction. (a) Initial pancreatogram. (b) Passage of a 0.025′′ guidewire. (c) Pancreaticogastrostomy tract dilation with 6 mm dilation balloon. (d) Placement of a 8 × 60 mm fully covered SEMS followed by 7 Fr × 7 cm single-pigtail plastic stents placement.

The echoendoscope is positioned either in the gastric body or duodenal bulb [13, 15]. The EUS-CP techniques are similar to those of biliary tree. During ductography, 1% methylene blue can be mixed in 1 : 4 ratio with full strength contrast. Methylene blue acts as guide to the location of pancreatic duct orifice in the small intestine. The guidewire is advanced antegrade towards the papilla for rendezvous or antegrade techniques. If not possible, the guidewire is advanced retrograde and looped in the pancreatic duct for transluminal approach (Figure 3).

## 4. Efficacy and Safety of EUS-CP

### 4.1. Definitions

All the published case reports and series were reviewed, and studies involving at least 5 patients were included for the present review. The data was separated into extrahepatic biliary, intrahepatic biliary, and pancreatic duct drainage. The technical success was defined as the decompression of the pancreatobiliary tree with placement of a stent and/or stone extraction [13]. The clinical success was defined as resolution of jaundice, pain relief [13], or major improvement of symptoms (like resolution of pancreatic fistula) [16]. Kahaleh et al. [17] measured mean pancreatic duct size, pain scores, and weight before and after the procedure as clinical success parameters.

### 4.2. Extrahepatic Biliary Tree

Table 2 presents the published data on extrahepatic biliary drainage. There are 21 studies involving 360 on extrahepatic biliary drainage via EUS-CP. The first case series of EUS-guided cholangiogram was reported by Wiersema et al. in 1996 [7]. Later, Giovannini et al. [18] reported the first case of transluminal stenting, followed by common bile duct stone removal by Püspök et al. [19]. Plastic stents were first placed transluminally to create a fistula, followed by stone removal in 3 weeks. Overall, the procedure was technically successful in 325/360 cases (90%; range 70–100%).The overall clinical success (if reported) was achieved in 254/258 (98%; range 60–100%). The overall complication rate was 51/360 cases (14%, range 0–47%). These included pneumoperitoneum, bile leak/peritonitis, hemobilia, bacteremia, pancreatitis, abdominal pain, and cardiopulmonary failure due to fluid overload [13].

### 4.3. Intrahepatic Biliary Tree

The published data for intrahepatic biliary drainage is listed in Table 3. There are 8 published studies involving 123 cases. The overall technical and clinical success rates were 109/23 (88.6%, range 44–100%) and 103/109 (94.5%, range 83–100%), respectively. The overall complication rate was 19/123 (15%, range 7.7–36%). These included pneumoperitoneum, cholangitis, bile leak, minor bleed, stent dysfunction (occlusion/migration), aspiration pneumonia, and even death from bile peritonitis due to stent migration in one patient [8].

The procedure timing was not reported by most of the studies. Kim et al. [20] reported a median procedure time of 19.5 minutes (range 14–35) for transluminal approach. In a case series of 6 patients with gastric bypass, the procedure time ranged from 66–78 minutes for antegrade and 100–144 minutes for rendezvous approaches. The stent types placed were both plastic (6–10 Fr, straight, single or double pigtail), and metal (8–10 mm, uncovered, partially fully covered). Either plastic or covered (partially/fully) metal stents were placed transluminally. Stent dysfunction in the form of either occlusion or migration was encountered more frequently with transluminal approach. Stent dysfunction was noted in 16 out of 55 patients (29%) in the study by Park do et al. [11], with reintervention successful in all patients with fully covered metal and in half with plastic stents. The mean stent patency was 133 days (range 18–433). 

### 4.4. Pancreatic Duct

Table 4 shows the published data on drainage on pancreatic duct via EUS-CP. There are 6 published studies involving 115 cases. Wiersema et al. [7] reported the first case on pancreatic ductography in 1996, followed by injection of methylene blue-contrast solution by DeWitt et al. in 2004 [21] to localize minor papilla in a patient with pancreas divisum. The largest pancreatic case series of 36 patients was reported by Tessier et al. in 2007 [22]. The overall technical and clinical success (if reported) rates were 90/115 (78%, range 48–91.7%) and 51/68 (75%, range 50–100%), respectively. The overall complication rates were 19/115 (16.5%, range 10–42.9%). These included pancreatitis (mild), abdominal pain, bleed, perforation, fever, severe pancreatitis, and even peripancreatic abscess [8]. Although there was no procedure-related mortality, severe complications (as previously mentioned) were noted with pancreatic drainage via EUS-CP. It is believed that EUS-guided pancreatic drainage is usually successful with dilated PD (≥4 mm), and complications are more likely with nondilated PD [8, 23]. The total procedure timings were reported by François et al. [24] in four cases: average 81.25 minutes (range 40–180). In the largest single-operator and single-session EUS-CP study by Shah et al. [9], the mean procedure time including failed ERCP was only 97 minutes (range 36–210) for both biliary and pancreatic cases. Pancreatic stent types used were plastic (5–10 Fr, straight, single or double pigtail). In the largest reported pancreatic series by Tessier et al. [22], stent dysfunction was noted in 22/36 (55%) cases. The median stent patency was 195 days (range 10–780).

## 5. Clinical Role of EUS-CP

At present, EUS-CP is increasingly been used at expert centers as an alternative to surgery or PTC. It should be considered in patients in whom ERCP has failed by an experienced endoscopist, and there is a need for pancreatobiliary drainage. Unlike PTC, EUS-CP can also be performed in patients with ascites [25]. However, only the left intrahepatic biliary tree can be accessed. For isolated right-sided biliary obstruction, PTC is still needed. Although suggested by Dhir et al. [26] in a retrospective nonrandomized study that EUS-guided rendezvous was a low-risk alternative to precut sphincterotomy for biliary cannulation, EUS-CP is a technically challenging procedure with a significant learning curve. The endoscopist should be proficient in both EUS and ERCP. Unlike pancreatic pseudocyst drainage, there is possibility of displacement between the puncture site and obstructed ducts with resultant failure and complications. The creation or dilation of fistula tract may be difficult due to fibrosis as in chronic pancreatitis. Care should be taken to avoid major vessels in the vicinity, like portal vein, hepatic artery, and splenic vessels. However, with increasing availability of endoscopists trained in both ERCP and EUS, the role of EUS-CP is likely to grow in clinical practice.

Same session EUS-CP as failed initial ERCP is practical and may result in avoidance of additional procedures. Combined duodenal and EUS-guided biliary stenting has also been shown to be practical [27]. Although nondilated ducts have been accessed, the puncture can be risky in such cases. The diameter of the working channel of the linear echoendoscopes is still limited, allowing small-caliber stents or delivery systems. There are no dedicated EUS-CP accessories. Commercially available one-step devices are needed. There are no studies directly comparing EUS-CP versus PTC.

## 6. Summary

EUS-CP is safe, efficacious, and a viable alternative to PTC or surgery in failed ERCP cases by an experienced endoscopist. It can be accomplished in one of the four ways: ductography, rendezvous, antegrade, or transluminal stenting. The overall technical and clinical success rates are around 90% for biliary tree and 70% for pancreatic duct drainage. The technical success rate is relatively low for pancreatic as compared to biliary cases. The overall EUS-CP complication rate was around 15%. Most of the complications are minor. However, severe complications can be encountered during pancreatic drainage. EUS-CP should be performed by an experienced endoscopist skilled in both EUS and ERCP. EUS-CP has a potential application in benign biliary cases. Same session EUS-CP as failed initial ERCP is practical and may result in avoidance of additional procedures. Since it tends to be a longer procedure, anesthesia support should be sought. Prophylactic antibiotics should be administered to all patients. Future research will be needed to improve instruments and accessories.

## Figures and Tables

**Table 1:** Instruments and accessories needed for EUS-CP.

Purpose	Devices
Echoendoscopes	Preferably therapeutic (>3 mm working channel):
	(i) GF-UCT140 (Olympus America Inc, Center valley, PA, USA): 3.7 mm
	(ii) EG-3870UTK (Pentax of America Inc, Montvale, NJ, USA): 3.8 mm

Puncture devices	(i) 19- or 22-gauge fine needle aspiration needles
	(ii) 19-gauge fistulotome
	(iii) 5 Fr needle knife

Guidewires	Long (450 or 480 cms):
	0.018 inch, 0.021 inch, 0.025 inch, or 0.035 inch

Dilation devices	Needed for transluminal and antegrade techniques:
	(i) 6–10 Fr bougie (SBDC; (Cook Medical Inc, Bloomington, IN, USA)
	(ii) 4–6 mm dilation balloon (Boston Scientific, Natick, MA, USA)
	(iii) ERCP 3.9–4.9 Fr sphincterotome (Boston Scientific, Natick, MA, USA)
	(iv) 5.5 Fr needle knife cautery (Boston Scientific, Natick, MA, USA)*
	(v) 6–8.5 Fr cystotome (EndoFlex, Voerde, Germany).

Stent types (as needed)	Biliary:
	Plastic (6–10 Fr; straight, single, or double pigtail)
	Metal (8–10 mm; uncovered, partially fully covered)^#^
	Pancreatic:
	Plastic (5–10 Fr; straight, single, or double pigtail)

EUS-CP: endoscopic-ultrasound-guided cholangiopancreatography. *Needle knife cautery is associated with increased risk of postprocedure complications. ^#^Either plastic or covered (partially/fully) metal stents are used for transluminal stenting.

**Table 2:** Published EUS-CP series on Extrahepatic biliary tree drainage (involving ≥5 patients).

Year	Author	*N*	Indication	Initial ERCP	Techniques	Technical success	Clinical success	Complication
1996	Wiersema et al. [7]	10	B	Both	D	7/10 (70%)	n/a	1/10 (10%)
2005	Püspök et al. [19]	5	M	Sb	T	5/5 (100%)	5/5 (100%)	No
2006	Kahaleh et al. [28]	10	Both	Sb	8 R; 2 T	9/10 (90%)	9/10 (90%)	3/9 (33%)
2008	Yamao et al. [29]	5	M	Sb	T	5/5 (100%)	5/5 (100%)	1/5 (20%)
2008	Tarantino et al. [30]	9	Both	Sb	4 T; 4 R; 1 D	9/9 (100%)	9/9 (100%)	No
2009	Maranki et al. [12]	14	Both	Sb (mostly)	8 R; 4 T	12/14 (86%)	12/12 (100%)	3/14 (21%)
2009	Brauer et al. [13]	12	Both	Sb	4 R; 4 T; 3 D	11/12 (92%)	11/11 (100%)	2/12 (16.7%)
2009	Horaguchi et al. [14]	8	M	Sb	T	8/8 (100%)	8/8 (100%)	1/8 (12.5%)
2010	Kim et al. [10]	15	Both	Sm (mostly)	R	12/15 (80%)	11/12 (91.7%)	2/15 (13.3%)
2010	Iwamuro et al. [27]	7	M	Sb	T	7/7 (100%)	7/7 (100%)	2/7 (28%)
2011	Siddiqui et al. [31]	8	M	Sb	T	8/8 (100%)	8/8 (100%)	2/8 (25%)
2011	Komaki et al. [32]	15	M	n/a	14 T; 1 R	15/15 (100%)	15/15 (100%)	7/15 (47%)
2011	Hara et al. [33]	18	M	n/a	T	17/18 (94%)	17/17 (100%)	3/18 (17%)
2011	Park do et al. [11]	26	Both	Sm	T	24/26 (92%)	22/24 (92%)	5/26 (19%)
2011	Ramírez-Luna et al. [34]	9	M	Sb	T	8/9 (89%)	8/8 (100%)	1/9 (11%)
2011	Fabbri et al. [35]	16	M	Sm	13 T; 3 R	12/16 (75%)	12/12 (100%)	1/16 (6.25%)
2012	Dhir et al. [26]	58	Both	Sm	R	57/58 (98.3%)	57/57 (100%)	2/58 (3.4%)
2012	Iwashita et al. [36]	31	Both	Sm	R	25/31 (81%)	25/25 (100%)	4//31 (13%)
2012	Kim et al. [20]	9	M	Sb	T	9/9 (100%)	9/9 (100%)	3/9 (33%)
2012	Shah* et al. [9]	70	Both	Sm	46 R; 20 A (or T); 2 D	60/70 (85.7%)	n/a	6/70 (8.5%)
2012	Maluf-Filho et al. [37]	5	M	Sm	T	5/5 (100%)	3/5 (60%)	2/5 (40%)

	Total	360			178 R; 141 T; 20 A; 16 D	325/360 (90%)	254/258 (98%)	51/360 (14%)

EUS-CP: endoscopic-ultrasound-guided cholangiopancreatography, *N*: number of patients, B: benign, M: malignant, Sb: subsequent day, Sm: same day/session, D: ductography, T: transluminal, R: rendezvous, A: antegrade, n/a: not applicable/mentioned. *The biliary tree was accessed at extra- as well as intrahepatic levels. However, the exact puncture site was not specified in the paper.

**Table 3:** Published EUS-CP series on intrahepatic (left) biliary tree drainage (involving ≥5 patients).

Year	Author	*N*	Indication	Initial ERCP	Techniques	Technical success	Clinical Success	Complication
2006	Kahaleh et al. [28]	13	Both	Sb	11 R*; 1 T	12/13 (92.3%)	12/12 (100%)	1/13 (7.7%)
2007	Bories et al. [38]	11	Both	Sb	T	10/11 (91%)	10/10 (100%)	4/11 (36%)
2007	Will et al. [7]	10^#^	Both	Sb	T	9/10 (90%)	8/9 (88.9%)	1/8 (12.5%)
2009	Maranki et al. [12]	35	Both	Sb (mostly)	24 R; 3 T; 2 A	29/35 (83%)	29/35 (83%)	5/35 (14.3%)
2009	Horaguchi et al. [14]	7	M	Sb	T	7/7 (100%)	6/7 (86%)	1/7 (14.3%)
2011	Park do et al. [11]	31	Both	Sm	T	31/31 (100%)	27/31 (87%)	5/31 (16%)
2011	Weilert et al. [39]	6	B	n/a	4 A; 2 R	6/6 (100%)	6/6 (100%)	1/6 (17%)
2012	Iwashita et al. [36]	9	Both	Sm	R	4/9 (44%)	4/4 (100%)	1/9 (11%)

	Total	123			63 T; 46 R; 6 A	109/123 (88.6%)	103/109 (94.5%)	19/123 (15%)

EUS-CP: endoscopic-ultrasound-guided cholangiopancreatography, *N*: number of patients, B: benign, M: malignant, Sb: subsequent day, Sm: same day/session, D: ductography, T: transluminal, R: rendezvous, A: antegrade, n/a: not applicable/mentioned. *In few cases stents might have been placed antegrade. ^#^10 interventions in 8 patients.

**Table 4:** Published EUS-CP series on pancreatic duct drainage (involving ≥5 patients).

Year	Author	*N*	Indication	Initial ERCP	Techniques	Technical success	Clinical success	Complications
2007	Will et al. [16]	12*	B	Sb	5 T; 4 R	8/12 (67%)	4/8 (50%)	6/14 (42.9%)
2007	Tessier et al. [22]	36	B	Sb	T	33/36 (91.7%)	25/36 (69%)	5/36 (13.8%)
2007	Kahaleh et al. [40]	13	B	Sb	5 R; 5 T	10/13 (77%)	10/10 (100%)	2/13 (15.4%)
2009	Brauer et al. [13]	8	B	Sb	4 T; 3 R	7/8 (88%)	4/8 (50%)	No
2010	Barkay et al. [8]	21	B	Sb	6 D (mb injection); 4 R	10/21 (48%)	8/8^#^ (100%)	2/21 (10%)
2012	Shah et al. [9]	25	B	Sm	10 A or T; 9 R; 3 D	22/25% (88%)	n/a	4/25 (16%)

	Total	115			46 T; 25 R; 10 A; 9 D	90/115 (78%)	51/68 (75%)	19/115 (16.5%)

EUS-CP: endoscopic-ultrasound-guided cholangiopancreatography, *N*: number of patients, B: benign, Sb: subsequent day, Sm: same day/session, D: ductography, T: transluminal, R: rendezvous, A: antegrade, mb: methylene blue, n/a: not applicable/mentioned. *14 attempts in 12 patients. ^#^Long-term data was available in 8 patients only.
